# APOE-ε4 Is Associated With Reduced Verbal Memory Performance and Higher Emotional, Cognitive, and Everyday Executive Function Symptoms Two Months After Mild Traumatic Brain Injury

**DOI:** 10.3389/fneur.2022.735206

**Published:** 2022-02-16

**Authors:** Torgeir Hellstrøm, Nada Andelic, Øyvor Øistensen Holthe, Eirik Helseth, Andres Server, Kristin Eiklid, Solrun Sigurdardottir

**Affiliations:** ^1^Department of Physical Medicine and Rehabilitation, Oslo University Hospital, Oslo, Norway; ^2^Research Center for Habilitation and Rehabilitation Models and Services (CHARM), Institute of Health and Society, University of Oslo, Oslo, Norway; ^3^Institute of Clinical Medicine, Faculty of Medicine, University of Oslo, Oslo, Norway; ^4^Department of Neurosurgery, Oslo University Hospital, Oslo, Norway; ^5^Section of Neuroradiology, Department of Radiology and Nuclear Medicine, Oslo University Hospital, Oslo, Norway; ^6^Department of Medical Genetic, Oslo University Hospital, Oslo, Norway; ^7^Centre for Rare Disorders, Oslo University Hospital, Oslo, Norway

**Keywords:** mild traumatic brain injury, *APOE*, learning, memory, affective symptoms

## Abstract

**Background:**

Substantial variance exists in outcomes after mild traumatic brain injury (MTBI), and these differences are not fully explained by injury characteristics or severity. Genetic factors are likely to play a role in this variance.

**Objectives:**

The aim of this study was to examine associations between the apolipoprotein (APOE)-ε4 allele and memory measures at two months post-MTBI and to evaluate whether subjective cognitive and affective symptoms were associated with APOE-ε4 status. Based on previous research, it was hypothesized that APOE-ε4 carriers would show poorer verbal memory performance compared to APOE-ε4 non-carriers.

**Methods:**

Neuropsychological data at two months post-injury and blood samples that could be used to assess *APOE* genotype were available for 134 patients with MTBI (mean age 39.2 years, 62% males, 37% APOE-ε4 carriers). All patients underwent computed tomography at hospital admission and magnetic resonance imaging four weeks post-injury.

**Results:**

The APOE-ε4 + status was associated with decreased immediate memory recall (*p* = 0.036; β = −0.10, 95% CI [−0.19, −0.01]). Emotional, cognitive, and everyday executive function symptoms at two months post-injury were significantly higher in APOE-ε4 carriers compared to non-carriers.

**Conclusion:**

The APOE-ε4+ allele has a negative effect on verbal memory and symptom burden two months after MTBI.

## Introduction

Individuals after a mild traumatic brain injury (MTBI) can display neuropsychological functioning ranging from normal to poor cognitive recovery, and subjective complaints can differ widely, from no symptoms to prolonged cognitive, emotional, and somatic symptoms (1). Several factors influence neurocognitive and functional outcomes, including demographic (e.g., age at injury, education), psychosocial (e.g., socioeconomic status, family status), premorbid functioning (e.g., intellectual ability, employment), and injury-related variables (e.g., severity, external cause of injury) (2–6). Over the last decade, an increasing number of studies have suggested that genetic polymorphisms, particularly the apolipoprotein (*APOE*) genotype, may play a pivotal role in the recovery process and help explain the heterogeneity in neurocognitive outcomes following MTBI (7–11). However, despite the growth in genetic and genomic research, the influence of genetic factors on neuropsychological performance, functional outcome, and neuroimaging findings following MTBI has yet to be fully investigated. It would be clinically meaningful to better understand heterogeneity in neurocognitive outcomes and the factors that contribute to increased risk of decline in memory over time in response to MTBI, as these deficits may reflect underlying brain dysfunction (12–14).

Findings from previous studies have shown that the *APOE* genotype is predictive of important outcomes after a TBI, including memory and processing speed, verbal episodic memory performance, level of global functioning, and mortality (8, 15–20). The product of the *APOE* gene serves many functions within the central nervous system, including lipid transportation and clearance, as well as maintenance of neuronal integrity and synapto-dendritic connections (21, 22). There are three common allelic variations of the *APOE* gene, ε2, ε3, and ε4, and these variants differ notably in their capacity for stimulating neurite outgrowth following a central nervous system insult (22, 23). Compared to the ε2 and ε3 alleles, the ε4 allele is detrimental in this process, as it inhibits neurite outgrowth, disrupts neuronal cytoskeleton, and magnifies amyloid beta accumulation (22, 23). Furthermore, APOE-ε4 is a well-known genetic risk factor for Alzheimer's disease and markedly exacerbates tau-mediated neurodegeneration (24), which can negatively affect cognitive functioning (22). Additionally, within the context of TBI, the ε4 allele has been linked with increased likelihood of cerebrovascular pathology (25), larger hematoma volume (26), and the presence of cerebral amyloid angiopathy (27).

The Glasgow Outcome Scale Extended (GOSE) is often used to measure the relationship between the APOE-ε4 allele and global patient outcomes following TBI. In a meta-analysis by Zhou et al. (15), APOE-ε4 carriage was associated with a higher risk of poor outcomes six months post-TBI. Another meta-analysis, by Zeng et al. (16), showed that the APOE-ε4 allele was associated with lower odds of a good prognosis, with a slightly larger effect size if only severe TBI was included. Further, a meta-analysis by McFayden et al. (17) demonstrated higher odds of a favorable functional outcome following TBI in APOE-ε4 non-carriers compared with APOE-ε4 carriers and individual homozygous for this allele. In a study of college athletes who sustained MTBI, Merritt et al. reported that APOE-ε4 carriers had significantly worse self-reported symptomatology at three months after injury, mostly within the physical and cognitive domains (28). However, in line with a study of professional fighters (29) and athletes (30), no clear effect of the APOE-ε4 allele on neuropsychological functioning was established (17). Several studies have reported a negative effect of the APOE-ε4 allele on memory (29, 30). For example, one study found that the APOE-ε4 allele may confer an increased risk of verbal memory impairment at six months after MTBI (7). Another study showed that the ε4 allele was associated with worse memory and processing speed as well as overall cognitive impairment among military veterans (8). Notably, however, a meta-analysis by Padgett et al. reported no significant differences in general cognitive or memory functions after TBI (31). A systematic review by Lawrence et al. (32) concluded that the effect of APOE-ε4 on TBI outcome was non-contributory in 14 studies (58.3%), hazardous in 9 (37.5%), and protective in 1 (4.2%). Most of the studies reported on fewer than 200 patients. Taken together, meta-analyses and systematic reviews have reported different effects of the APOE-ε4 on TBI outcomes in studies describing outcomes for diverse populations of patients with mild, moderate, or severe TBI, as well as patients with different characteristics (pediatric, adolescent, adult, older adult) and in different settings in which the TBI was sustained (civilian, sports-related, military).

In the present study, we evaluated a homogenous sample of adult patients with MTBI. All patients were hospitalized due to the injury and followed up at two months post-injury. The aim was to examine the associations between the APOE-ε4 allele and five verbal memory measures at two months after injury. Further, we assessed whether subjective cognitive and affective symptoms at two months post-injury were associated with APOE-ε4 status, as well as group differences between ε4 (+) carriers and non-carriers of ε4 (-) determined using neuroimaging with the use of acute computed tomography (CT) and magnetic resonance imaging (MRI) at four weeks post-injury. Based on previous literature, we hypothesized that APOE-ε4 would have a negative impact on verbal memory and that APOE-ε4 carriers with MTBI would experience more cognitive and affective symptoms compared to APOE-ε4 non-carriers.

## Materials and Methods

### Participants and Procedure

Patients with an acute MTBI who were admitted to the Oslo University Hospital, Oslo, Norway, between September 2011 and September 2013 were included in a prospective cohort study. Patients aged 16–65 years with a recent (<24 h) history of head trauma (hospitalization with ICD-10 diagnosis S06.0–S06.9) resulting in a loss of consciousness (LOC) <30 min, post-traumatic amnesia (PTA) <24 h, and a Glasgow Coma Scale (GCS) score between 13 and 15 were included. MTBI was defined using criteria from the American Congress of Rehabilitation Medicine (33). Exclusion criteria were confirmed diagnosis of severe mental illness in medical records (e.g., schizophrenia or bipolar disorder), progressive neurologic disease, ICD-10 diagnosis of substance dependence, contraindications for MRI, and lack of Norwegian language skills.

### Biospecimen and Genotyping Procedures

The variants in exon four in the *APOE* gene (GenBank: NM_000041)—that is, c.388T > C and c.526C > T—were analyzed by polymerase chain reaction and Sanger sequencing in DNA extracted from peripheral leukocytes. Primers were designed using Primer3Plus (Bioin-formatics, Arlington, VA, USA) (34) and the polymerase chain reaction products were purified and Sanger sequenced using an ABI 3730xl DNA analyzer and an ABI BigDye terminator cycle-sequencing kit v3.1 (Thermo Fisher Scientific, Waltham, MA, USA). Sequences were analyzed with the DNA Sequencing Analysis software program (version 5.1; Applied Biosystems, Foster City, CA, USA) and the SeqScape software program (version 2.7; Thermo Fisher Scientific).

### Patient Groups: APOE-ε4(+) and APOE-ε4(–)

For the current analyses, patients with available neuropsychological data at two months post-injury and APOE blood samples were included. Of the 176 participants included at 2 months (35), complete data were available for 138, but 4 patients were excluded due to lack of motivation (see below). Fifty of the patients included (37%) were APOE-ε4(+) carriers and 84 (63%) were APOE-ε4(–) non-carriers.

Information regarding age, sex, and education level was obtained from a clinical interview. The GCS (36) was used to assess the conscious state, and total scores on this scale can be between 3 (showing no response) and 15 (alert and well-oriented). The presence and duration of PTA and LOC were determined based on medical records and classified into no PTA vs. yes/unknown and no LOC vs. yes/unknown. External cause of injury was obtained from medical records and classified as traffic accidents, falls, violence, or other. The CT taken at admission to hospital (CT acute) on clinical indication was used to assess intracranial injuries.

### MRI Data Acquisition and Analysis

3T MRI (GE Signa HDxt, GE Medical Systems, Milwaukee, WI, USA) data was obtained four weeks post-injury using two different head coils (Head/Neck/Spine [HNS] and 8HRBRAIN). MRI scans were performed using a 3T whole-body MRI system. The protocol included a 3D Fast Spoiled Gradient Echo (FSPGR) T1-weighted sequence used for morphometric assessments (repetition time msec/echo time msec/inversion time msec, 7,8744/2.96/450; flip angle, 12°; and spatial resolution, 1 × 1.3 × 1.2 mm). Acquisition parameters were optimized for increased gray/white matter contrast. In addition, a T2-weighted sequence and a T2 susceptibility-weighted angiography (SWAN) sequence were performed to depict hemorrhagic or other lesions. No major scanner upgrade occurred during the study period. MRI scans were evaluated for gross pathologies by a neuroradiologist.

### Neuropsychological Assessment

Participants were assessed by one neuropsychologist without prior knowledge of the participants'* APOE* genotypes. In this study, the raw scores of five sub-tests of the California Verbal Learning Test-II (CVLT-II) (37) were of interest to assess learning and memory: recall of 16 words presented over five trials (Immediate Recall Trials 1–5), two short-delay recalls (Short-Delay Free Recall and Short-Delay Cued Recall), and two delay recalls of the word list after 20 min (Long-Delay Free Recall and Long-Delay Cued Recall). The CVLT-II is recommended for use in the TBI population and is reported to have excellent reliability and validity (38). The CVLT-II was also used in a similar study by Yue et al. (7), making it possible to compare our results with their findings.

The Rey Fifteen-Item Test, which is used to assess symptom validity, was used to measure lack of motivation. In this test, the participant is shown 15 items for 10 s and is then requested to draw what they recall. In this study, a lack of motivation was defined as a score of ≤ 9. Four patients were excluded due to low scores.

### Self-Reported Outcomes

The Behavioral Rating Inventory of Executive Function-Adult Version (BRIEF-A) (39) consists of 75 items that assess nine aspects of executive functioning in everyday life, with responses scored from 1 (never) to 3 (often). The BRIEF-A consists of a composite index score, the Global Executive Composite (GEC), and two sub-index scores; the Behavioral Regulation Index (BRI) and the Metacognition Index (MI). Raw scores are transformed into age-corrected T-scores.

The Rivermead Post-Concussion Symptoms Questionnaire (RPQ) assesses frequently occurring symptoms in the cognitive, emotional, and somatic domains (40). Individuals were asked to rate symptoms over the past 24 h on a scale from 0 (not experienced at all) to 4 (a severe problem). The items from each sub-scale were summed to a total score, with ratings of 1 excluded. The sub-scale scores (RPQ somatic, emotional and cognitive) and the total RPQ score were used in this study.

The Hospital Anxiety and Depression Scale (HADS) (41) has two subscales (Anxiety and Depression), each consisting of seven items rated on a 4-point scale from 0 (no symptom) to 3 (severe symptom). The validity and reliability of the HADS has been established in patients with TBI (42, 43). Both the sub-scales scores (range 0–21) and the total HADS score (range 0–42) were used in the analyses.

### Functional Outcome

The GOSE measures global function, including independence; work, social, and leisure activities; and participation in social life (44). It is an 8-point ordinal scale reflecting good recovery (7, 8), moderate (5, 6) and severe (3, 4) disability, vegetative state (2), and death (1).

### Statistical Analysis

Descriptive statistical analyses were performed using SPSS for Windows, version 26 (SPSS Inc., Chicago, IL, USA). Sample characteristics are presented as the group mean and standard deviation (SD). Differences between patient groups on continuous variables were tested using Student's *t*–test. The Chi-square test was used to detect group differences in categorical variables. Statistical power was calculated by the program G^*^power (45). The sample size of 134 would give a statistical power of 0.88 at α level of 0.05, with medium effect size (*F*^2^ = 0.15) and nine predictors.

Tests of normality and skewness indicated that the CVLT-II, age at the time of the two-month assessment, and length of stay (LOS) at the emergency hospital deviated from a normal distribution. Logarithmic transformations of these variables were therefore used in the regression analyses, with lower scores on the CVLT-II reflecting better performance, lower scores on age indicating lower age, and lower scores in LOS indicating fewer days at the emergency hospital.

Hierarchical multiple regressions were conducted to assess the relationship between the five CVLT-II outcome measures and the APOE-ε4 genotype, using a bootstrap with 1,000 replications. The regression analyses are presented as β coefficients, with 95% confidence intervals (95% CI) for each predictor, *p*-values, explained variance (R^2^), and the ΔR^2^. For the first step, the predictor variables of age at the two-month assessment, education (0 = over 12 years, 1 = 12 years or less), gender (0 = male, 1 = female), employment (0 = employed, 1 = unemployed), LOS at the emergency hospital, LOC (0 = no, 1 = yes), PTA (0 = no amnesia, 1 = amnesia), and brain CT and MRI brain scans (0 = uncomplicated MTBI without CT/MRI findings, 1 = complicated MTBI with CT/MRI findings) were included. Genotype was added in the second step [0 = APOE-ε4(+), 1 = APOE-ε4(–)]. Findings with a two-tailed *p*-value of < 0.05 were considered statistically significant.

## Results

### Patients and Injury Characteristics

The overall sample (*n* = 134 MTBI patients) was predominantly male (62%) and had a mean age of 39.2 years (SD 14.4). When stratified by APOE-ε4 status, the ε4(+) and the ε4(–) groups did not differ significantly in any demographic or clinical variables (Table 1). The majority of the injuries occurred due to falls (39%), followed by traffic injuries (38%). The majority of the patients had a GCS score of 15 (72%), and there were 68 uncomplicated and 66 complicated MTBIs. None of the patients had undergone intracranial surgery. Psychiatric or medical comorbidities were extracted and checked in medical records; 11 patients (8%) had anxiety, 11 patients (8%) had depression, 10% were diagnosed with headaches and six patients (4%) had cardiac diseases. Twenty percent of the 30 patients with comorbidity in our study had two or more comorbidities. Univariate regression analyses did not reveal any significant associations between the abovementioned comorbidities with APOE-ε4 status or outcome measures (CVLT-II). Hospital length of stay ranged from 1 to 31 days (SD 3.3) and there were no significant difference between the two APOE-ε4 groups (*p* = 0.39). A small proportion of patients received rehabilitation (6%) and physiotherapy (10%) during the two months post injury. Thirty-six patients (27%) with persistent symptoms were offered clinical assessments by an outpatient rehabilitation team after the two months follow-up at the Oslo University hospital.

**Table 1:** Demographics and injury-related characteristics stratified by the APOE-ε4 status groups.

**Variables**	**MTBI sample** **(*N* = 134)**	**APOE** **ε4(–)** **(*N* = 84)**	**APOE** **ε4(+)** **(*N* = 50)**	***p*-value**
Age (years)	39.2 (14.4)	40.7 (14.3)	37.0 (14.5)	0.16
Gender - Male - Female	83 (62%) 51 (38%)	51 (61%) 33 (39%)	32 (64%) 18 (36%)	0.71
Education - Less than 12 years - Over 12 years	63 (47%) 71 (53%)	38 (45%) 46 (55%)	25 (50%) 25 (50%)	0.59
Mechanism of injury - Traffic accidents - Falls - Violence - Other	51 (38%) 52 (39%) 17 (13%) 14 (10%)	31 (37%) 36 (43%) 10 (12%) 7 (8%)	20 (40%) 16 (32%) 7 (14%) 7 (14%)	0.55
GCS score - 13–14 - 15	38 (28%) 96 (72%)	26 (31%) 58 (69%)	12 (24%) 38 (76%)	0.39
LOC - No - Yes/unknown	26 (19%) 108 (81%)	19 (23%) 65 (77%)	7 (14%) 43 (86%)	0.22
PTA (n, %) - No amnesia - Yes/unknown	13 (10%) 121 (90%)	10 (12%) 74 (88%)	3 (6%) 47 (94%)	0.26
Length of hospital stay (days)	2.5 (3.3)	2,29 (2,32)	2,88 (4,52)	0.39
MRI and CT pathology - No - Yes	68 (51%) 66 (49%)	43 (51%) 41 (49%)	25 (50%) 25 (50%)	0.89
Site of injury on MRI (*n* = 131) - Frontal - Temporal - Frontotemporal - Parietal - Occipital	29 (22%) 23 (17%) 11 (8%) 13 (10%) 7 (5%)	14 (11%) 16 (12%) 5 (4%) 9 (7%) 6 (5%)	15 (11%) 7 (5%) 6 (5%) 4 (3%) 1(0.8%)	0.09 0.40 0.33 0.77 0.25

*APOE, apolipoprotein E, with plus sign (+) denoting ε4 carriers and minus sign (–) denoting ε4 non-carriers; GCS, Glasgow Coma Scale; LOC, loss of consciousness; PTA, post-traumatic amnesia; CT, computed tomography; MRI, magnetic resonance imaging*.

*p-values: T-test for continuous variables; Chi square and Fischer's exact test for categorical variables. Data are presented as means (SD) or frequencies (n, %)*.

### APOE-ε4 and Memory Outcomes at Two Months Post-injury

The results of the multiple regressions are presented in Table 2 and show the predictors' standardized coefficients (β), their 95% CIs, and statistically significant effects. The APOE-ε4 status was significantly associated with the Immediate Recall Trials 1–5 (*p* = 0.036) and improved the model in the second step (ΔR^2^ = +0.023). The APOE-ε4(+) genotype was associated with a decrease in immediate memory recall (β = −0.10, 95% CI [−0.19, −0.01]). Lower age (β = 0.52, 95% CI [0.22, 0.82]) and more education (β = 0.24, 95% CI [0.13, 0.34]) were strongly associated with increased immediate recall (*p*-values < 0.001). No other variables emerged as significant predictors.

**Table 2:** Multivariable regression analyses of APOE-ε4 genotype and verbal memory subscales (California Verbal Learning Test-II) at two months post-injury.

**Variable**	**β (95% CI)**	***p*-value**
Immediate Recall Trial 1–5	(*R^2^* = 0.458; *ΔR^2^* = +0.023)	
APOE-ε4(+) Age (years) Education (over 12 years) Gender (male) Employment (yes) Emergency hospital stay LOC (yes/unknown) PTA (yes/unknown) CT/MRI pathology (yes)	−0.10 [−0.19, −0.01] 0.52 [0.22, 0.82] 0.24 [0.13, 0.34] −0.04 [−0.15, 0.07] −0.05 [−0.17, 0.09] −0.15 [−0.36, 0.02] −0.02 [−0.15, 0.12] 0.06 [−0.13, 0.29] −0.03 [−0.14, 0.09]	0.036 <0.001 <0.001 0.513 0.532 0.119 0.798 0.541 0.666
Short-Delay Free Recall	(*R^2^* =0.383; *ΔR^2^* = +0.020)	
APOE-ε4(+) Age (years) Education (over 12 years) Gender (male) Employment (yes) Emergency hospital stay LOC (yes/unknown) PTA (yes/unknown) CT/MRI pathology (yes)	−0.09 [−0.18, 0.01] 0.34 [0.05, 0.61] 0.20 [0.10, 0.29] 0.02 [−0.07, 0.12] −0.06 [−0.20, 0.07] −0.08 [−0.23, 0.07] 0.05 [−0.09, 0.19] 0.06 [−0.16, 0.26] 0.04 [−0.07, 0.15]	0.085 0.025 <0.001 0.664 0.399 0.282 0.450 0.568 0.455
Short-Delay Cued Recall	(*R^2^* =0.398; *ΔR^2^* = +0.007)	
APOE-ε4(+) Age (years) Education (over 12 years) Gender (male) Employment (yes) Emergency hospital stay LOC (yes/unknown) PTA (yes/unknown) CT/MRI pathology (yes)	−0.05 [−0.16, 0.04] 0.34 [0.07, 0.67] 0.21 [0.10, 0.31] −0.03 [−0.13, 0.08] −0.01 [−0.15, 0.14] −0.16 [−0.38, 0.01] 0.02 [−0.11, 0.17] 0.01 [−0.19, 0.20] 0.05 [−0.05, 0.16]	0.336 0.029 <0.001 0.547 0.976 0.101 0.749 0.914 0.370
Long-Delay Free Recall	(*R^2^* =0.407; *ΔR^2^* = +0.015)	
APOE-ε4(+) Age (years) Education (over 12 years) Gender (male) Employment (yes) Emergency hospital stay LOC (yes/unknown) PTA (yes/unknown) CT/MRI pathology (yes)	−0.08 [−0.17, 0.02] 0.40 [0.09, 0.71] 0.20 [0.10, 0.30] −0.03 [−0.13, 0.07] −0.02 [−0.17, 0.13] −0.11 [−0.29, 0.06] 0.07 [−0.07, 0.21] 0.10 [−0.09, 0.29] 0.01 [−0.10, 0.12]	0.119 0.016 0.001 0.525 0.783 0.235 0.364 0.295 0.892
Long-Delay Cued Recall	(*R^2^* =0.429; *ΔR^2^* = +0.012)	
APOE-ε4(+) Age (years) Education (over 12 years) Gender (male) Employment (yes) Emergency hospital stay LOC (yes/unknown) PTA (yes/unknown) CT/MRI pathology (yes)	−0.07 [−0.18, 0.03] 0.45 [0.16, 0.76] 0.21 [0.11, 0.32] −0.01 [−0.11, 0.10] 0.01 [−0.15, 0.15] −0.13 [−0.29, 0.03] 0.07 [−0.08, 0.23] 0.06 [−0.14, 0.24] 0.03 [−0.08, 0.14]	0.165 0.001 0.003 0.866 0.960 0.153 0.377 0.512 0.560

*APOE, apolipoprotein E; CVLT-II, California Verbal Learning Test-2nd edition; β, standardized effect size; CI, confidence interval; LOC, loss of consciousness; PTA, post-traumatic amnesia; CT, computed tomography; MRI, magnetic resonance imaging*.

*Bootstrapped regression analyses were used to assess the association of APOE ε4 genotype on memory performance*.

For the Short-Delay Free Recall, a near-significant effect of APOE-ε4 status was found (*p* = 0.085) and improved the model (ΔR^2^= +0.020). Lower age (β = 0.34, 95% CI [0.05, 0.61]) and more education (β = 0.20, 95% CI [0.10, 0.29]) were associated with better performance on the Short-Delay Free Recall (*p* = 0.025 and *p* < 0.001, respectively). No other variables emerged as significant predictors. For the other three CVLT-II models, only age and education emerged as significant predictors.

### APOE-ε4, Self-Reported Outcomes, and Global Functioning at Two Months Post-injury

The APOE-ε4(+) genotype was generally associated with higher symptom pressure (see Table 3). Specifically, significantly higher scores were observed in the APOE-ε4(+) group on dimensions of post-concussive symptoms, as represented by emotional and cognitive symptoms (RPQ) and the Global Executive Composite and Metacognition and Behavioral Regulation indices of the BRIEF-A. There were no other significant differences between the two groups in anxiety or depression. Moreover, the two groups reported equal function on the GOSE.

**Table 3:** Self-reported outcome measures according to APOE-ε4 status group.

**Variables** **2 months post-injury**	**Overall** **(*N* = 134)**	**APOE-ε4(–)** **(*N* = 84)**	**APOE-ε4(+)** **(*N* = 50)**	***p*-value**
RPQ-total, mean (SD)	11.5 (12.5)	10.2 (12.4)	13.6 (12.6)	0.12
RPQ-somatic, mean (SD)	5.3 (6.0)	5.2 (6.3)	5.4 (5.6)	0.83
RPQ-emotional, mean (SD)	2.9 (4.3)	2.3 (4.0)	4.0 (4.7)	0.02
RPQ-cognitive, mean (SD)	3.3 (3.7)	2.7 (3.4)	4.2 (3.9)	0.02
BRIEF-A Global Executive Composite T-score	48.9 (11.3)	47.2 (11.5)	51.6 (10.5)	0.03
BRIEF-A Metacognition index T-score	50.0 (11.4)	48.3 (11.7)	52.9 (10.5)	0.02
BRIEF-A Behavioral Regulation Index T-score	47.8 (10.5)	46.4 (10.2)	50.0 (10.7)	0.05
HAD-total, mean (SD)	7.51 (6.39)	7.30 (6,56)	7.88 (6,15)	0.61
HAD-anxiety, mean (SD)	4.71 (3.83)	4.55 (3.81)	4.98 (3.88)	0.53
HAD-depression, mean (SD)	2.81 (3.23)	2.75 (3.52)	2.90 (2.70)	0.78
GOSE-total, mean (SD)	6.80 (0.91)	6.88 (0.92)	6.68 (0.89)	0.22

*APOE, apolipoprotein E, with plus sign (+) denoting ε4 carriers and minus sign (–) denoting ε4 non-carriers; RPQ, Rivermead Post-Concussion Symptoms Questionnaire; BRIEF-A, Behavioral Rating Inventory of Executive Function-Adult Version; HADS, Hospital Anxiety and Depression Scale; GOSE, Glasgow Outcome Scale Extended*.

### APOE-ε4 and Acute Findings on Brain Scans

Significantly more epidural hematomas on the acute CT were observed in the APOE-ε4(+) group (see Supplementary Table 1). On the MRI at four weeks after MTBI (see Supplementary Table 2), there were no significant differences in type of intracranial injury between the two groups. Diffuse axial injuries (DAI) in the APOE-ε4(+) group were more frequent, but this difference was not significant (*p* = 0.07).

## Discussion

The primary aim of the present study was to examine the association between the ε4 allele of the *APOE* gene and verbal memory two months after MTBI in a civilian population. Consistent with our hypothesis, APOE-ε4(+) was associated with decreased immediate memory recall at two months post-injury. In general, age and education had significant impacts on all five subtests of verbal memory (CVLT-II). Interestingly, in this study, the injury characteristics were not associated with verbal memory performance. Thus, memory deficits in MTBI do not seem to occur solely based on age and education or injury severity. Our study confirms the finding of a study by Yue et al. (7) that APOE-ε4 may confer an increased risk of impairment of verbal memory in the first months after injury. Recent studies on cognitive functions in patients with MTBI have found that cognitive deficits were associated with APOE-ε4 status in this patient group (7, 8, 20). In addition, in a study of athletes with concussions, Merritt et al. (30) demonstrated greater neurocognitive variability in APOE-ε4 carriers (+) compared to non-carriers. Such findings are in line with those of previous studies demonstrating an association between *APOE* genotype and cognitive reduction in TBI populations (8, 32, 46, 47). Conversely, other studies concluded that APOE-ε4 carriers with TBI performed at a cognitive level similar to that of APOE-ε4 non-carriers (31, 48–50). These discrepancies might be attributable to differences in study design and methodologies applied. Our sample size was comparable to that of many previous studies on APOE-ε4 and TBI in systematic reviews (32) and larger than the sample size in the study by Yue et al. (7).

Though more research is needed, our results suggest that APOE-ε4 increased the likelihood of cognitive dysfunction the first months after trauma even in patients with mild TBIs. Although speculative, it is possible that possession of one copy of the APOE-ε4 allele might result in increased risk of cognitive decline due to altered repair mechanisms following neurotrauma or exacerbate underlying MTBI-related cognitive impairment. The precise mechanisms responsible for the negative effects of the APOE-ε4 allele on cognitive functioning following MTBI are not well-established. However, research supports the notion that, compared with the APOE ε2 and ε3 alleles, the ε4 allele of the *APOE* gene possesses a number of properties that may hinder recovery following neurological injury (51). For example, APOE-ε4 genotype status was associated with increased cerebral edema and brain inflammation (52), which might result in slower brain recovery that in turn negatively influences cognitive performance early after MTBI. Individuals from a Norwegian population (53) have a lower frequency of APOE4 (APOE4 allele frequency of about 20%) than in our study (37%). It is possible that having the allele could represent a risk factor for suffering mTBI but this has to be explored in futures studies.

As MTBI varies greatly in clinical presentation, the shift to objective findings rather than subjective symptom complaints may offer a new approach for clinicians to understand patients' needs early after injury and determine when treatment changes are required (e.g., prevention of memory decline). Thus, finding a predictable marker of worse cognitive outcome would offer advantages in assessment of prognosis, allowing resources to be focused on the immediate aftermath of injury in those patients deemed vulnerable.

This study also investigated the occurrence of subjective cognitive and affective symptoms in MTBI patients at two months after injury. Everyday executive function complaints (BRIEF-A) and cognitive and emotional symptoms (RPQ) were more commonly reported by the APOE-ε4(+) carriers compared to non-carriers, which may suggest that subjective cognitive symptoms are a reflection of the immediate memory recall deficits. However, no significant between-group differences were found in ratings of depressive and anxiety symptoms (HADS) or functional outcome (GOSE) at two months post-injury. Our data suggest that there may be ways to both identify the neurocognitive and behavioral characteristics associated with MTBI and use APOE-ε4 status to better manage the MTBI in APOE-ε4 carriers. To our knowledge, these everyday executive changes reported by the APOE-ε4(+) carriers early after injury are novel findings in the MTBI literature.

With respect to intracranial injuries, the results showed a significantly higher proportion of epidural hematomas on acute CT in the group of APOE-ε4 carriers. Although APOE-ε4 carriers had a higher prevalence of epidural hematomas on acute CT, there was not a higher prevalence of contusions or other intracranial injuries. This is line with the findings of Liaquat et al., who showed that the *APOE* genotype did not affect the risk of suffering an intracranial hematoma after head injury (26). Epidural hematomas occur more frequently among younger men (54), and although the difference was not significant, the mean age of the APOE-ε4(+) group was 3.7 years younger than that of the APOE-ε4(–) group. However, we cannot rule out that the significantly higher proportion of epidural hematomas on acute CT in the group of APOE-ε4 carriers represents a Type 1 error from the relatively small sample size.

The present study has several strengths, including that it had a sample of patients with MTBI with no significant differences in patients' characteristics between the APOE-ε4 carriers and non-carriers, as well as a prospective design. To our knowledge, this study is the first to combine data on a reliable memory test, subjective cognitive complaints, depressive symptoms, global functioning, and CT/MRI brain scans in groups of MTBI patients divided by *APOE* genotype. The relatively homogenous nature of our sample provides an advantageous setting in which to examine the relationship between the *APOE* genotype and neurocognitive functioning in patients with MTBI. The data presented here suggest that memory and everyday executive problems should be addressed early in MTBI patients with the APOE-ε4 allele. However, this study has some limitations. Our findings are not generalizable to children, adolescents, and older adults or to patients with premorbid psychiatric history and drug abuse, as these patient groups were excluded from this study. There were no blast injuries included. Moreover, the number of participants included in our study is smaller than the recommended sample size for a genetic study (55). On the other hand, our sample size was large enough to detect medium effects sizes with acceptable power but a larger sample would be needed to have statistical power to detect small effect sizes. Therefore, this study might be underpowered to detect genetic differences in neurocognitive outcomes. A further limitation is the lack of a control group without TBI for comparison. Thus, we cannot rule out that the presence of APOE-ε4 is associated with decreased verbal memory independent of a mTBI. Another limitation includes not assessing the patients' cognitive recovery over time, particularly when undertaking cognitive screening in APOE-ε4(+) carriers. These patients may have significantly improved over time as described in a previous TBI study (56) and/or shown poorer outcome (17, 18). Finally, additional larger studies are required to determine if there is correlates of brain injury location with PCS symptoms or neuropsychological scores amongst APOE-ε4(+) carriers. Parenchymal contusions or axonal injuries to regions of eloquence may also impact outcome and represent an area of important and imminent future research.

## Conclusions

Our findings suggest that carrying the APOE-ε4 genotype has a negative effect on verbal memory two months after MTBI. Future investigations would benefit from larger sample sizes to increase statistical power as well as from using a longitudinal design. More research would be needed to assess the potential moderating influence of other genes implicated in neural recovery or protection. More detailed investigations into the pathophysiological consequences related to MTBI biomarkers may yield further insights and more useful MTBI biomarkers for clinical practice.

## Data Availability Statement

The datasets presented in this study can be found in online repositories. The names of the repository/repositories and accession number(s) can be found below: https://www.ncbi.nlm.nih.gov/genbank/, NM_000041.

## Ethics Statement

The studies involving human participants were reviewed and approved by the Norwegian Regional Committee for Medical Research Ethics (2010/1899) (Oslo, Norway). The patients/participants provided their written informed consent to participate in this study. Written informed consent was obtained from the individual(s) for the publication of any potentially identifiable images or data included in this article.

## Author Contributions

TH, NA, and SS: conceptualization, drafting of the manuscript, and statistical analysis. TH, NA, ØH, EH, AS, KE, and SS: analysis, interpretation of data, and critical revision of the manuscript for important intellectual content. All authors have read and agreed to the published version of the manuscript.

## Conflict of Interest

The authors declare that the research was conducted in the absence of any commercial or financial relationships that could be construed as a potential conflict of interest.

## Publisher's Note

All claims expressed in this article are solely those of the authors and do not necessarily represent those of their affiliated organizations, or those of the publisher, the editors and the reviewers. Any product that may be evaluated in this article, or claim that may be made by its manufacturer, is not guaranteed or endorsed by the publisher.
