# Supplementary material for: APOE-ε4 Is Associated With Reduced Verbal Memory Performance and Higher Emotional, Cognitive, and Everyday Executive Function Symptoms Two Months After Mild Traumatic Brain Injury
Source: Front Neurol. 2022 Feb 16;13:735206. doi: 10.3389/fneur.2022.735206 (PMC8888909; doi:10.3389/fneur.2022.735206)
Supplement: Supplementary file 1 [file Table_1.DOCX]

| **Variables** | **APOE ε4 (-)**  **(N=82)** | **APOE ε4 (+)**  **(N=48)** | **p-value** |
| --- | --- | --- | --- |
| Contusion yes/no | 17/65 | 6/42 | 0.24 |
| EDH yes/no | 2/80 | 7/41 | **0.01** |
| SDH yes/no | 12/70 | 4/44 | 0.29 |
| SAH yes/no | 21/61 | 9/39 | 0.37 |
| ICH yes/no | 2/80 | 4/44 | 0.12 |
| Skull fracture yes/no | 21/61 | 16/32 | 0.35 |

Notes. EDH= epidural hematoma; SDH= subdural hematoma; SAH= subarachnoid hemorrhage; ICH= intracranial hemorrhage.

p-values: Chi square test for categorical variables.
