# Supplementary material for: APOE-ε4 Is Associated With Reduced Verbal Memory Performance and Higher Emotional, Cognitive, and Everyday Executive Function Symptoms Two Months After Mild Traumatic Brain Injury
Source: Front Neurol. 2022 Feb 16;13:735206. doi: 10.3389/fneur.2022.735206 (PMC8888909; doi:10.3389/fneur.2022.735206)
Supplement: Supplementary file 2 [file Table_2.DOCX]

Additional Table 2. Magnetic resonance imaging findings at 4 weeks post-injury between APOE-ε4 status groups.

| **Variables**  4 weeks | **APOE ε4 (-)**  **(N=81)** | **APOE ε4 (+)**  **(N=50)** | **p-value** |
| --- | --- | --- | --- |
| Contusions yes/no | 24/57 | 13/37 | 0.65 |
| DAI yes/no | 3/78 | 6/44 | 0.07 |
| EDH yes/no | 2/79 | 3/47 | 0.31 |
| SDH yes/no | 2/79 | 3/47 | 0.31 |
| SAH yes/no | 2/79 | 1/49 | 0.86 |
| ICH yes/no | 2/79 | 3/47 | 0.31 |

Notes. DAI = diffuse axonal injury; EDH = epidural hematoma; SDH = subdural hematoma; SAH = subarachnoid hemorrhage; ICH = intracranial hemorrhage.

p-values: Chi-square test for categorical variables.
